# Sixth scientific and international meeting in Shisong captures new healthcare challenges

**DOI:** 10.11604/pamj.2017.27.175.11397

**Published:** 2017-07-05

**Authors:** Appolonia Budzee, Jean Claude Ambassa, Mve Mvondo Charles, Fanka Marcel, Jacques Cabral Tantchou Tchoumi

**Affiliations:** 1 Saint Elizabeth Catholic General Hospital Shisong, Cardiac Centre, PO box: 08 Kumbo, North West region, Cameroon

**Keywords:** Scientific day, Cardiac centre, Shisong

## Abstract

The rapid-changing scientific and technological landscape is triggered and in turn, warrants continuous scientific research. The Shisong Cardiac Center, instead of being contented with just repeating theses that have been proven elsewhere, is determined to contribute to the scientific community worldwide its experiences and findings which are both universal and unique. The Cardiac Center of St. Elizabeth Catholic General Hospital, Shisong, in the North West Region of Cameroon, was inaugurated in 2009 by Health Minister, Mr. Andre MAMA FOUDA. Its capacity is able to serve all of Central Africa, where such services are rare.

## Meeting Report

**Figure 1:**
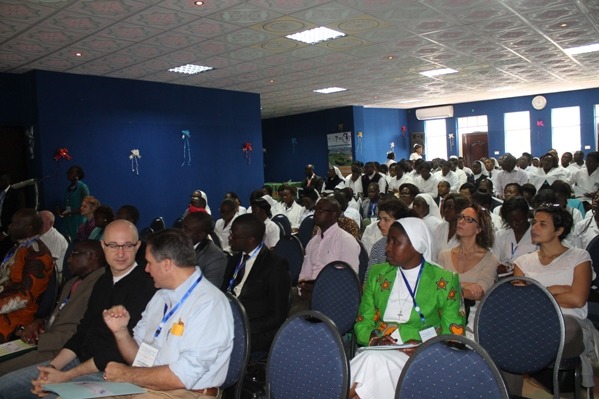
The attendees at the 6th scientific and international meeting

The day started with a speech given by Reverend Sister Aldrine KINYUY, Director of St. Elizabeth Catholic General Hospital, welcoming the population. St. Elizabeth Catholic General Hospital is centre of excellence in the management of all patients coming with various types of diseases. “With the ever growing challenges in diagnostic and cardiac surgical interventions over the years, this scientific meeting has thus been designed to provide an innovative and comprehensive overview of the challenges in cardiology and other disciplines faced within our operating milieu. She called on participants (Figure 1) to be open minded in sharing and gaining knowledge from the in order to be helped to overcome the challenges being faced over the years. She equally noted that “the coincidental inauguration of the Conference Hall of the Cardiac Centre [1] today has been realized to serve us during occasions like this”. She finally thanked the organizing committee for efforts done in making the day a success. The chief medical officer appreciated the sacrifices of the doctors coming from far and stressed the need of having a forum regularly. He appreciated the stature of moderators and panelists, selected from various specialties, as testifying the importance and relevance of the conference. In his turn, he expressed an ardent wish that everyone in our respective areas of competence in the medical field would make good use of the conference so as to better manage and treat “patients for whom we have been called and to whom we administer”. Dr Tantchou Cabral TJ presented the research and scientific committee members who worked hard to plan for the day. Presentations were divided into three sections, medical, cardiac and miscellaneous.

**Figure 2:**
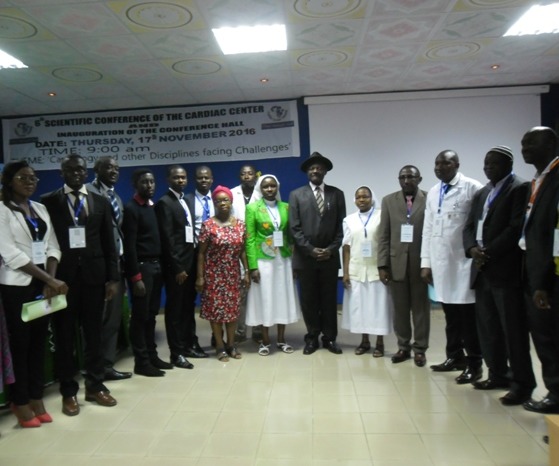
The doctors of St. Elizabeth catholic general hospital and others with the senior divisional officer of Bui

**Figure 3:**
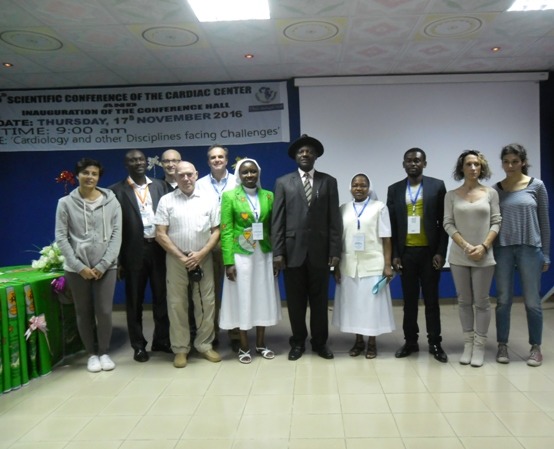
The international delegation with the senior divisional officer of Bui

Messages from the first presenter were based on our daily practice, informative, challenging and very sound. “Cardiomyopathies are one of the diseases in the Cardiac Centre with high mortality”, declare Mrs. NJOJHAM Odette in the opening presentation. Mrs. Electa Lybarfe enumerated the causes of poor compliance and mortality of patients suffering from tuberculosis, making it obvious that tuberculosis treatment is still a big challenge in St. Elizabeth Catholic General Hospital despite the National Program alleviating financial cost for the population. The interventional management of congenital heart diseases; precisely patent ductus arteriosus, is a reality in Shisong Cardiac Centre, of which Dr Ambassa Jean Claude (Figure 2) presented the challenges his team faces during his procedures. “To have managed the 76 cases in the catheterization laboratory with very good result, collaboration with all the actors: patient, staff, doctor was very important and crucial”, he urged. Dr Giamberti alessandro (Figure 3) continued with the updates in pediatric cardiology while the resident cardiovascular surgeon Dr. Mvondo Charles presented the results of surgical management of children with post rheumatic valvular lesions. The District Medical Officer, in his speech, focused participants’ attention on the role of the Cardiac Centre in saving lives of patients with cardiovascular diseases in Cameroon and the whole central African Region. The meeting ended at 5.25 pm with the closing remarks of the manager of the Cardiac Centre. One of the highlights of the day was at 9h30 am, the inauguration of the modern conference hall of the Cardiac Centre by his Excellency NZEKI Theophile, the Senior Divisional Officer for Bui. The conference hall is a huge investment of 25 million francs cfa, with a capacity of 250 places, completely soundproof as the standard imposes: 80 decibel, a system of renewing air, consisting of air conditioning and extractors. The hall is equipped with an automatic system for video-projection, enabling the projection of case of live cases from various areas of the Centre, and a lighting system designed for the comfort and relaxation of participants. The clean rest rooms are equipped with detector switches for maximize hygiene and minimize running cost and a modern technical room. The Senior Divisional Officer of Bui appreciated this beautiful piece, present in his jurisdiction area. Shisong Cardiac Centre [2] is the only private cardiosurgical Center in Central Africa, equipped with state-of-the- art technologies and prepared to offer a wide range of cardiology services including diagnosis and treatment of congenital heart defects, coronary artery disease, valvular heart disease and electrophysiology. It manages non-invasive cardiology; that is, diagnostic testing for patients with suspected cardiac problems through tests such as electrocardiography, holter, stress test, three-dimensional colour, pulsed and continuous doppler-echocardiography. It also offers both diagnostic and interventional catheterism in a hemodynamic laboratory as well as implantation of permanent single and dual-chamber pace makers and defibrillators. Open-heart surgeries with extracorporeal circulation are also regular at the Centre. The Project is jointly sponsored by the *Tertiary Sisters of St. Francis*, the Association *Bambini Cardiopatici nel Mondo and Cuore Fratello Association*in Milan (Italy) [3]. Cost of constructing and equipping the Center was about 4 billion FCFA (800 millions USD).

The Cardiac Centre Shisong is committed to sharing our learning experiences as a contribution for the improvement of healthcare especially in Sub Sahara Africa. There is a huge need for health research to support contextually relevant health service and policy solutions to better the health of populations in sub-Saharan Africa. This need contrasts with the very timid engagement of healthcare practitioners in research in the region. The Scientific and Research Committee of the Cardiac Center was created by its General Manager, Rev. Sr. Jethro Nkenglefac in 2011 to fulfill the scientific orientation of the CCS. The main goals of the SRC are: to participate to international randomized controlled clinical trials, perform clinical trials, organize update classes and scientific days, follow-up students on internship, publication of scientific papers in different famous international and national journals. Since its creation, 25 manuscripts have been published in international and national journals, 15 students performed their internship in Shisong Cardiac Centre. Moreover a Procedure and Policy Manual of the CCS has been elaborated, Saturday update and discussion classes are going on normally and the 6^th^ Scientific international day has been organized with a great success. It is hoped that this event will enhance clinical research and the dissemination of research findings to improve evidence-based clinical practice in the Country so that our particular healthcare challenges can be checked.

## Competing interest

The authors declare no competing interest.
